# Nimotuzumab combined with radiotherapy+/- chemotherapy for definitive treatment of locally advanced squamous cell carcinoma of head and neck: a metanalysis of randomized controlled trials

**DOI:** 10.3389/fonc.2024.1380428

**Published:** 2024-06-13

**Authors:** Meng Guan, Dan Zhang, Yue Zhao, Mingdi Mao, Kang Shen, Xia Wang, Chun Bi

**Affiliations:** ^1^ Department of Oncology, The First People's Hospital of Jiangxia District, Wuhan City and Union Jiangnan Hospital, Huazhong University of Science and Technology, Wuhan, China; ^2^ The First Clinical College of Xuzhou Medical University, Xuzhou, China; ^3^ Siyang Hospital Oncology Department, Suqian, China

**Keywords:** locally advanced head and neck squamous cell carcinoma, nimotuzumab, chemoradiotherapy, radiotherapy, meta-analysis

## Abstract

**Objectives:**

To assess the efficacy and safety of nimotuzumab in combination with radiotherapy or chemoradiotherapy for locally advanced head and neck squamous cell carcinoma.

**Methods:**

Systematic searches were performed on PubMed, Web of Science, Embase, Cochrane Library, China National Knowledge Infrastructure, China Biomedical Medicine, Wanfang, VIP databases. Seven eligible randomized controlled trials (n = 1012) were selected through rigorous inclusion and exclusion criteria.

**Results:**

A total of 1012 cases were included. including 508 (50.2%) in the nimotuzumab combination treatment group; There were 504 cases (49.8%) in the control group. The results of meta-analysis showed that the overall survival (Hazard Ratio [HR]=0.75, 95% Confidence Interval [CI]: 0.62-0.90, P<0.05), progression-free survival (HR=0.69, 95% CI: 0.54-0.87, P<0.05), complete response rate (Risk Ratio [RR]=1.52, 95% CI: 1.24-1.86, P<0.05), and objective response rate (RR=1.32, 95% CI: 1.17-1.48, P<0.05) were significantly improved in the nimotuzumab combination treatment group compared with the control group. In terms of the incidence of adverse effects, only the incidence of rash was the nimotuzumab combination group higher than in the treatment alone group, and there was no significant difference between the remaining adverse reactions (neutropenia, anemia, nausea/vomiting, mucositis, dermatitis, dysphagia).

**Conclusion:**

Nimotuzumab combined with radiotherapy or chemoradiotherapy is more effective than radiotherapy alone or chemoradiotherapy in locally advanced squamous cell carcinoma of the head and neck, and the safety profile is controllable. Therefore, the addition of nimotuzumab to treatment is expected to be an effective treatment option for this disease. However, more prospective randomized controlled trials are needed to fully explore the effectiveness of this treatment in patients with locally advanced head and neck squamous cell carcinoma.

**Systematic Review Registration:**

identifier PROSPERO (CRD: 42022383313).

## 1 Introduction

Squamous cell carcinoma of the head and neck (SCCHN) is a common type of malignancy. In the US, an estimated 54000 new cases and 11230 deaths in 2022 (1). Management of SCCHN depends on the anatomical location and stage of the disease. In the case of locally advanced disease, radical radiotherapy combined with platinum-based therapy is standard therapy unless additional chemotherapy is not required because of poor physical condition or comorbidities (2).

Studies have shown that, Anti-epidermal growth factor receptor(EGFR) protein expression is seen in 90% or more of the SCCHN cases (3). A recent multivariate analysis demonstrated that over expression of EGFR in subjects with Locally advanced squamous cell carcinoma of head and neck(LA-SCCHN) was associated with early relapses, lower disease-free, and overall survival (4–6). EGFR overexpression is also resistant to radiation therapy and chemotherapeutic agents, leading to treatment failure (7). These suggests that EGFR monoclonal antibody (MAB) combined with radiotherapy (RT) or chemoradiotherapy (CRT) may benefit patients with LA-SCCHN.

Nimotuzumab is a humanized anti-EGFR MAB, which was obtained in 1996 after genetic modification of the Cetuximab parental murine molecule ior egf/r3 (8). Studies have shown that, Nimotuzumab can inhibit the downstream cascade of EGFR signaling by specifically binding to the extracellular domain of EGFR, Blocking of EGFR, signaling, To benefit cancer patients (9); It can also activate NK cells to exert the cytotoxic effect of antibody-dependent cells (10); Simultaneously activated NK cells induce the maturation of dendritic cell and tumor specific CD8 + T cells, To restore the HLA-I expression on the tumor cells, Thus, the reversal of the EGFR-mediated tumor immune evasion mechanism (4). In addition, the affinity of nimotuzumab to EGFR is medium, and it will selectively target tumor cells with high expression of receptors, but it has less binding with normal tissues such as skin and mucosa, resulting in low toxic response (11). Therefore, nimotuzumab can be used effectively and repeatedly for a long time. It has been approved by many countries for the treatment of nasopharyngeal carcinoma, pediatric and adult glioma, and advanced esophageal cancer, etc. (2, 12, 13). Since 2009, several studies have reported that nimotuzumab combined with RT/CRT is used for the treatment of LA-SCCHN. However, compared with CRT or RT alone, whether the combined use can improve the efficacy of LA-SCCHN and whether it can increase adverse reactions has not been evaluated by systematic meta-analysis. Therefore, this meta-analysis of randomized controlled trials (RCT) aims to systematically evaluate the efficacy and safety of nimotuzumab combined with RT/CRT in the treatment of LA-SCCHN and provide more evidence for the treatment of LA-SCCHN.

## 2 Materials and methods

This meta-analysis strictly adheres to the PRISMA statement and is registered on PROSPERO (CRD: 42022383313). All analyses were conducted based on previously published studies; thus, no ethical approval and patient consent are required.

### 2.1 Inclusion criteria

1. The pathological diagnosis was SCCHN;
2. The clinical TNM stage was stage III or IVa, IVb;
3. To compare the efficacy and safety of nimotuzumab combined with CRT/RT and CRT/RT alone;
4. Have one or more results: including overall survival (OS), progression free survival (PFS), complete response rate (CRR), objective response rate (ORR), disease control rate (DCR) or adverse events; OS, the time from diagnosis of LA-SCCHN to death due to any reason; PFS, the time from diagnosis of LA-SCCHN to tumor progression or death from any cause in any aspect; The efficacy of the two treatment groups was evaluated using the criteria for evaluating the efficacy of solid tumors (RECIST): CRR, all target lesions disappeared; PRR, the sum of the total diameter of the target lesion decreased by at least 30%; PD, the sum of the total diameters of the target lesions increased by at least 20%, or new lesions appeared; SD, taking the minimum value of the sum of total diameters as a reference, the reduction of target lesions did not reach PR, and the increase did not reach the level of PD; ORR, the proportion of patients with tumors shrinking to a certain extent and staying for a certain time, including CRR and PRR cases; DCR, the proportion of patients with disease remission (CRR+PRR) and stable SD after treatment, that is, the proportion of patients without disease progression PD;
5. The study design was a randomized controlled trial (RCT).

### 2.2 Exclusion criteria

1. Articles are *in vitro* tests, reviews, meta-analysis, conference summaries or case reports;
2. The study was a no randomized controlled trial (nRCT) or observational study;
3. Including studies on patients with nasopharyngeal carcinoma;
4. Articles that cannot be used, such as poor report quality and unclear data description and etc.

### 2.3 Search strategy

In the PubMed, Web of Science, Embase, Cochrane Library, China National Knowledge Infrastructure(CNKI), China Biomedical Medicine(CBM), Wanfang, VIP databases, two researchers(MG and CB) respectively used terms such as head and neck cancer, head and neck squamous cell cancer, oral cancer, tongue cancer, oropharyngeal cancer, laryngeal cancer, hypopharyngeal cancer, nimotuzumab, and their synonyms for systematic retrieval. At the same time, we also reviewed conference abstracts of the unpublished articles and searched the references list of relevant studies to find other potential studies. The last retrieval time was November 23, 2020. According to the inclusion and exclusion criteria, the literatures that meet these criteria are strictly selected.

### 2.4 Data extraction and quality assessment

Two investigators (MG and CB) independently extracted relevant data from all eligible studies, including first author, year of publication, country, study design, and number of cases, stage, age, treatment scheme, dose of nimotuzumab, dose of concurrent chemotherapy drugs. And, we also extracted the main outcome indicators: OS and PFS (Only the HR and 95% CI of both are extracted. If they cannot be extracted from the text, the method proposed by Tierney (14) is used to calculate these statistical variables using the available numerical data.), secondary outcome measures: CRR, ORR, DCR, and incidence rate of adverse events, including neutropenia, anemia, thrombocytopenia, nausea/vomiting, oral mucositis, radiation dermatitis, rash, dysphagia. Two researchers (YZ and FT) independently evaluated the quality of the finally included literature according to the Review Manager 5.3 software provided by the Cochrane collaboration network, including random sequence generation (selection bias), allocation concealment (selection bias), blinding of participants and personnel (performance bias), incomplete outcome data (attrition bias), selective reporting (reporting bias) and other biases, and assessing each risk of bias as high, low, or unclear risk (15). Any differences arising in the process of data extraction and quality assessment shall be jointly discussed and resolved by researchers.

### 2.5 Statistical analysis

Statistical analysis of the extracted study data was performed using Review Manager 5.3 and Stata15.0. OS and PFS are reported by hazard ratios (HRs) and 95% confidence intervals (CIs); The rest of the results were reported by risk ratios (RRs) and 95% confidence intervals (CIs). The P values were all bilateral, and statistical significance was set at P<0.05. We used the Cochrane Q test and I^2^ statistic to assess the heterogeneity of all included studies. If the heterogeneity was not significant (P>0.1, I^2^<50.0%), a fixed-effects model was used; otherwise, a random-effects model was used. We used funnel plot and Egger test to evaluate potential publication bias. P>0.05 indicates that there is no potential publication bias.

## 3 Results

### 3.1 Study characteristics

**Figure 1:**
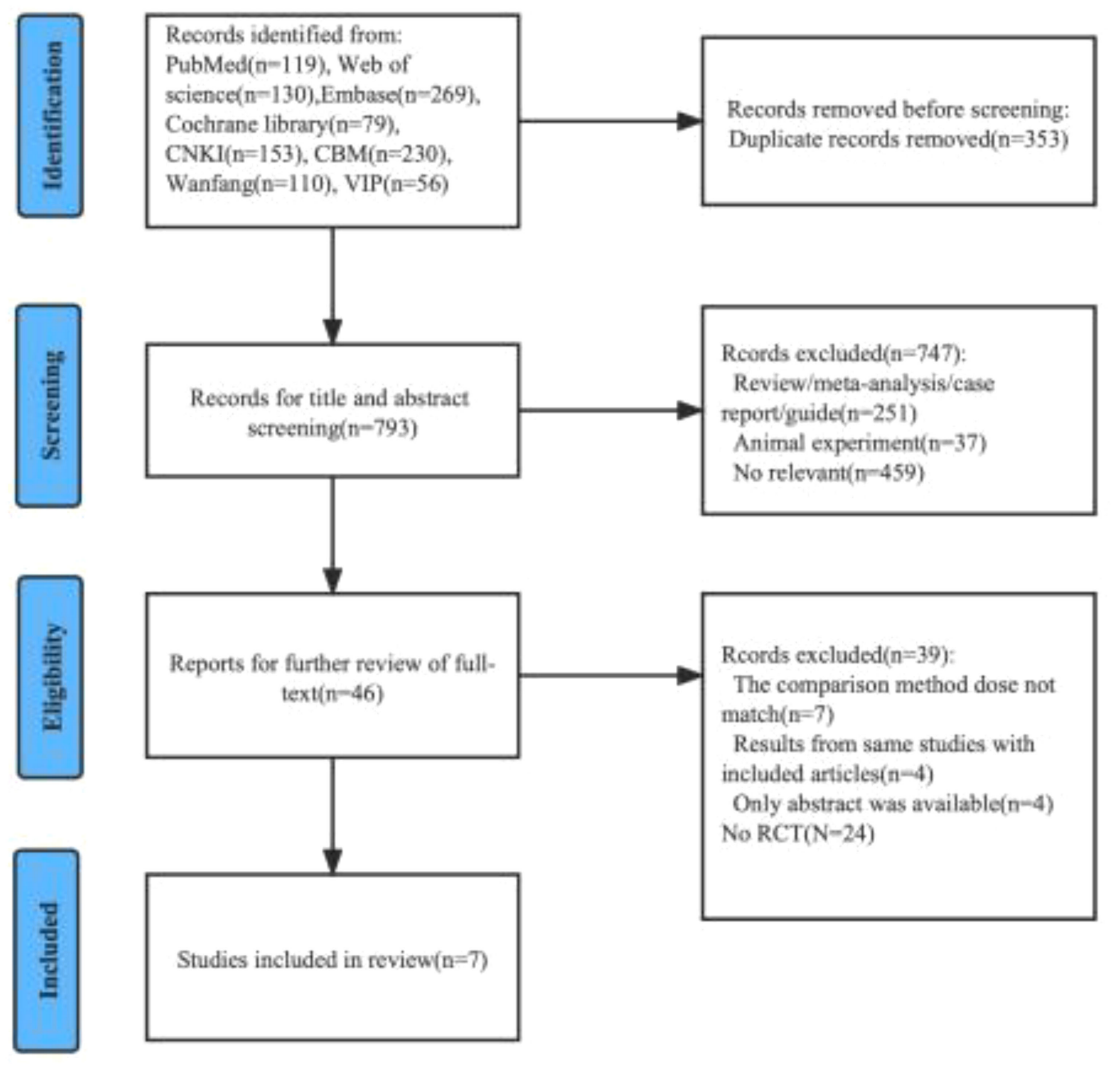
Flow chart of the literature selection process.

We preliminarily identified 1146 relevant articles from the database. After removing 352 duplicate literatures, we evaluated and screened the title, abstract or full text according to strict inclusion and exclusion criteria, and finally included 7 eligible RCT for meta-analysis (16–22). The detailed screening procedure is shown in **Figure 1**. 1012 cases were included, including 508 cases (50.2%) in the experimental group (nimotuzumab + CRT/RT) and 504 cases (49.8%) in the control group (CRT/RT). All patients treated with CRT had cisplatin-based concurrent chemoradiotherapy, and all patients treated with RT alone were considered as unsuitable cases for chemotherapy. The experimental group was treated with nimotuzumab according to the treatment protocol of the control group. The base information of the included studies is shown in **Table 1**.

**Table 1:** Characteristics of studies included.

Author	Year	Country	Study period	Study design	Number of cases (Exp/Con)	Stage	Age/Years		Treatment scheme		Nimotuzumab dose	Concurrent chemotherapy drugs dose
							Exp	Con	Exp	Con		
Kumar,A	2019	India	NA	RCT	15/14	III、IVa、IVb	55.0±8.29	56.0±6.5	CRT+nimotuzumab	CRT	200mg/week/6weeks	cisplain 40-50mg/m2 /week/6weeks
Patil,V.M	2019	India	2012-2018	RCT	268/268	III、IVa、IVb(AJCC 7th)	55(20-73)	54(26-77)	CRT+nimotuzumab	CRT	200mg/week/6weeks	cisplain 30mg/m2/week/6weeks
Reddy,B.K	2014	India	2004.09-2005.07	RCT	23/23	III、IVa(T1-T4a,N0-N2)	50(27-65)	55(30-68)	CRT+nimotuzumab	CRT	200mg/week/6weeks	cisplain 50mg/week/6weeks
Reddy,B.K	2014	India	2004.09-2005.07	RCT	23/23	III、IVa(T1-T4a,N0-N2)	60(43-70)	60(40-70)	RT+nimotuzumab	RT	200mg/week/6weeks	–
Rodriguez,M.O	2010	Cuba	2002.07-2007.02	RCT	54/52	III、IV	59.48	65.88	RT+nimotuzumab	RT+placebo	200mg/week/6weeks	placebo 200mg/week/6weeks
Thai Hoa,N.T	2020	Vietnam	2010.06-2013.06	RCT	43/44	III、IVa、IVb(AJCC)	NA	NA	CRT+nimotuzumab	CRT	200mg/week/6weeks	cisplain 30mg/m2/week/6weeks
Wu HX	2020	China	2016.03-2019.03	RCT	40/40	III、IVa	57±4.32	56.35±4.36	CRT+nimotuzumab	CRT	200mg/week/6weeks	cisplain 100mg/m2,q3w
Zhu QX	2021	China	2016.01-2019.01	RCT	42/40	III、IVa、IVb	54.8±3.1	53.5±2.7	CRT+nimotuzumab	CRT	200mg/week/6weeks	cisplain 75mg/m2,q3w

RCT, randomized controlled trial; CRT, chemoradiotherapy; RT, radiotherapy; NA, Missing value.

### 3.2 Quality assessment of the included literature

**Figure 2:**
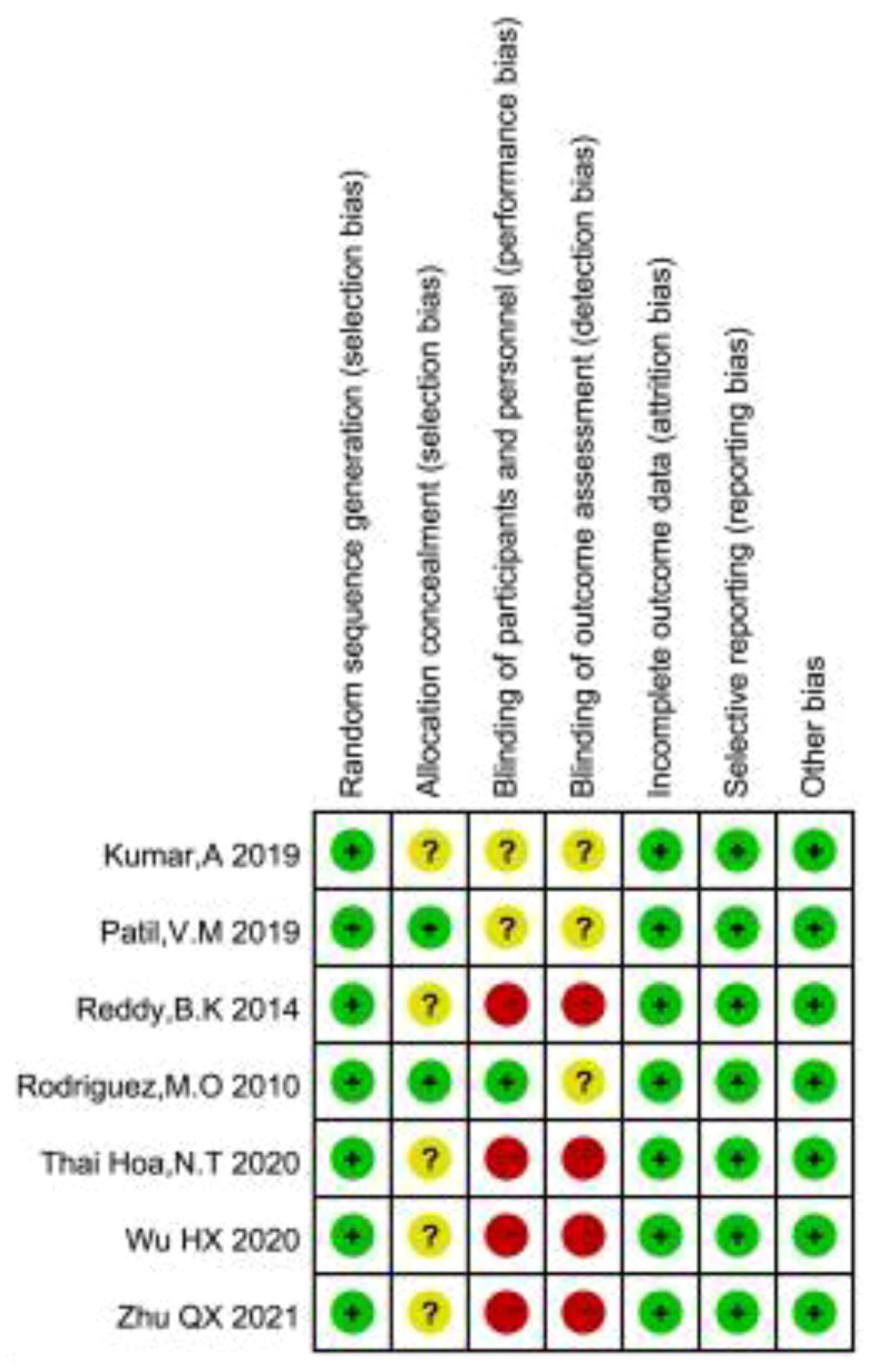
Risk of bias summary.

**Figure 3:**
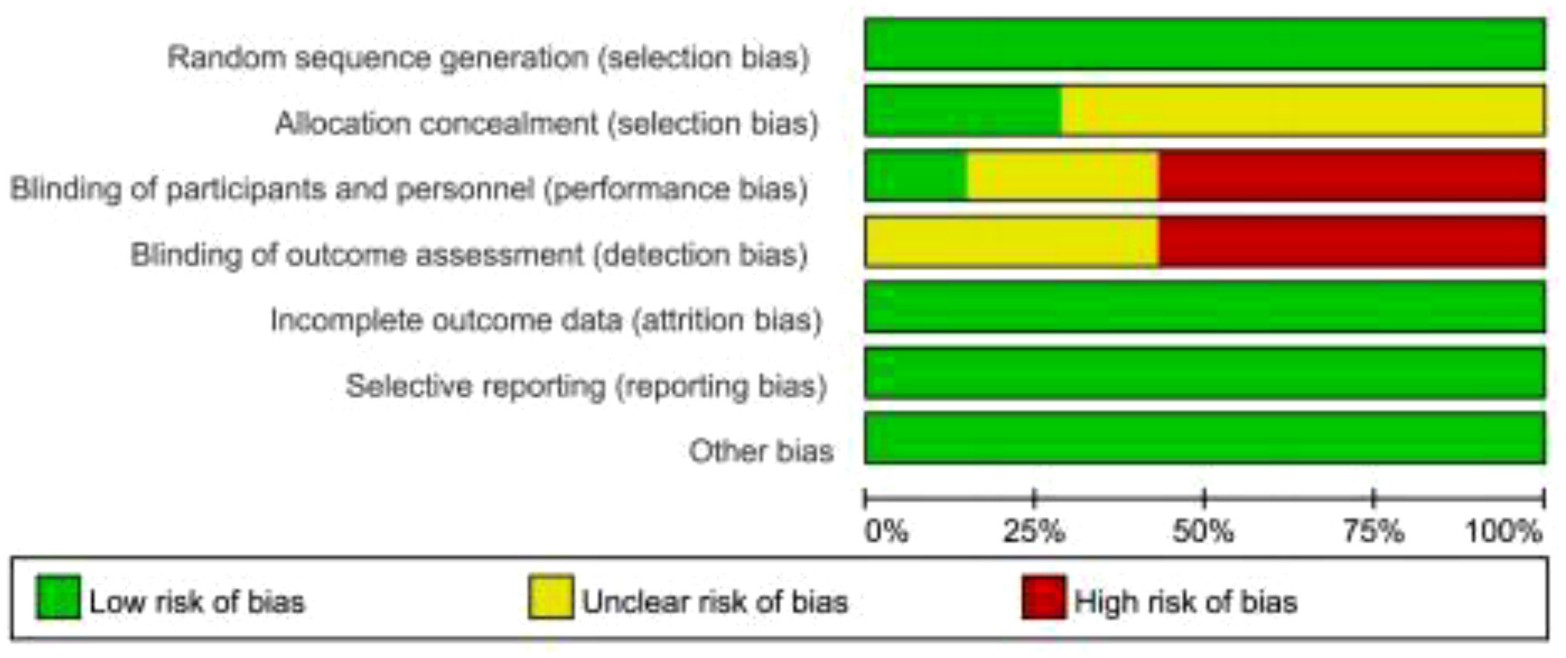
Risk of bias summary.

As shown in **Figures 2**, **3**, we assessed the quality of the final included studies using the tool of the Cochrane Collaboration. All the final included studies were RCT. Five studies (16–18, 20, 22) provided the randomization methods, and the remaining two studies (19, 21) did not describe any specific randomization method. Two studies (17, 20) provided information indicating that the allocation scheme was hidden. Four studies (16, 19, 21, 22) obtained informed consent from all included patients and were considered non-blind; one study (20) was double-blind; and the other studies (17, 18) did not mention the information related to blindness. The data and reports of the results included in the study were complete, without selective reporting or other biases. Overall, the risk of bias was low in all the studies included in this meta-analysis.

### 3.3 Main results

#### 3.3.1 OS

**Figure 4:**
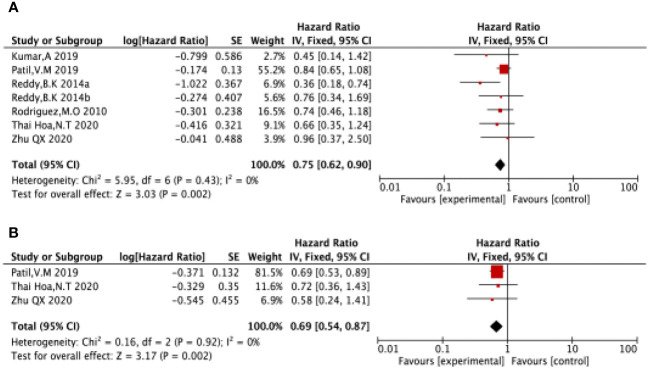
Forest plot for OS **(A)**, PFS **(B)** of nimotuzumab combined with RT/CRT group and RT/CRT alone group. OS, overall survival; PFS, progression free survival; CI, confidence interval; HR, hazard ratio.

Six of the included studies (17–21) reported OS in LA-SCCHN patients with no heterogeneity between studies (P = 0.43, I^2 = ^0%), and therefore, we used a fixed-effects model for our analysis. The results showed a pooled HR = 0.75 (95%CI 0.62-0.90). Suggested that patients receiving nimotuzumab plus CRT/RT had longer OS in LA-SCCHN compared to the CRT/RT group (P=0.002 <0.05, **Figure 4A**).

#### 3.3.2 PFS

Three of the included studies (16, 17, 21) reported PFS in LA-SCCHN patients with no heterogeneity between studies (P = 0.92, I^2 = ^0%); and therefore, we used a fixed-effects model for our analysis. The results showed HR = 0.69 (95%CI 0.54-0.87). Suggested that patients treated with nimotuzumab plus CRT/RT had better PFS compared to the CRT/RT group (P=0.002 <0.05 **Figure 4B**).

### 3.4 Secondary outcome

#### 3.4.1 CRR

**Figure 5:**
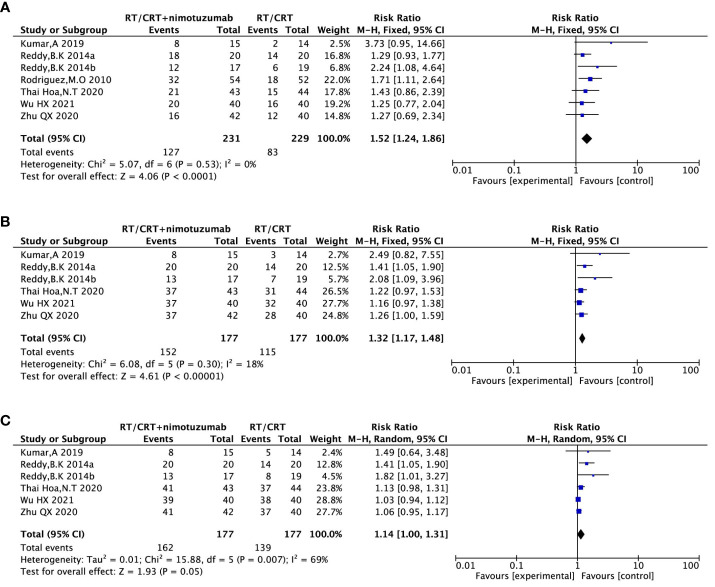
Forest plot for CRR **(A)**, ORR **(B)**, DCR **(C)** of nimotuzumab combined with RT/CRT group and RT/CRT alone group. CRR, complete remission rate; ORR, objective response rate; DCR, disease control rate; CI, confidence interval; RR, risk ratio.

Six (16, 18–22) (460 patients) in the included study reported CRRs for LA-SCCHN patients. There was no heterogeneity between the studies (P = 0.53, I^2 = ^0%); Therefore, we used a fixed-effects model for the analysis. The results showed that CRR was significantly higher in nimotuzumab plus CRT/RT group compared to the CRT/RT group (RR = 1.52,95%CI:1.24-1.86, P <0.05/RT) (**Figure 5A**).

#### 3.4.2 ORR

Five (16, 18, 19, 21, 22) (354 patients) in the included study reported the ORR for the LA-SCCHN patients. There was no heterogeneity between the studies (P = 0.30, I^2 = ^18%); Therefore, we used a fixed-effects model for our analysis. The results showed that the ORR was significantly higher in nimotuzumab and CRT/RT group compared to the CRT/RT group (RR = 1.32, 95%CI:1.17-1.48, P <0.05) (**Figure 5B**).

#### 3.4.3 DCR

Five (16, 18, 19, 21, 22) (354 patients) in the included study reported DCRs for LA-SCCHN patients. By heterogeneity, P = 0.007 <0.1, I^2 = ^69%> 50%, suggesting that there was heterogeneity among the literature selected for this study, so a random effect model was applied for the analysis. Results showed that this result was not statistically significant. (RR = 1.14,95%CI:1.00-1.31, P =0.05) (**Figure 5C**).

#### 3.4.4 Adverse reactions

**Figure 6:**
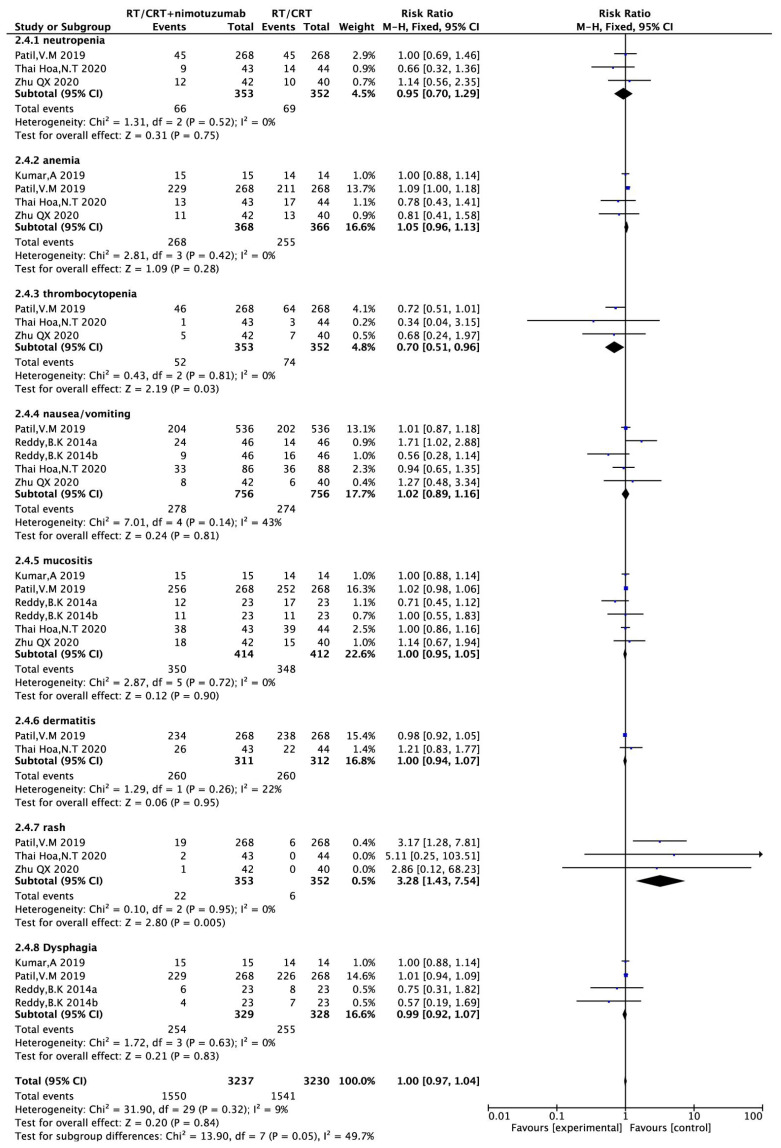
Forest plot for adverse reactions of nimotuzumab combined with RT/CRT group and RT/CRT alone group. CI, confidence interval; RR, risk ratio.

Three (16, 17, 21) (705 patients) reported neutrophilic reduction, four (16–18, 21) (734 patients) reported anemia, three studies (16, 17, 21) (705 patients) reported thrombocytopenia, four (16, 17, 19, 21) (797 patients) reported nausea/vomiting, five (16–19, 21) (826 patients) reported oral mucositis, two (16, 17) (623 patients) reported radiation dermatitis, three (16, 17, 21) (705 patients) reported rash, and three (17–19) (657 patients) reported dysphagia. There was no heterogeneity across studies (P> 0.1, I^2^<50%), so a fixed-effects model was used for the analysis. The results showed that the incidence of rash was higher in the nimotuzumab combination group than in the control group (RR = 3.28,95%CI:1.43-7.54, P =0.005 <0.05). The incidence of platelets in nimotuzumab was lower than in the control group, and the difference was statistically significant (RR=0.70, 95%CI:0.51-0.96, P=0.03<0.05). There were no significant differences in the incidence of the remaining neutrophilia, anemia, nausea/vomiting, oral mucositis, radiation rash, and dysphagia. (RR=0.95, 95%CI:0.70-1.29, P=0.75>0.05)、(RR=1。05, 95%CI:0.96-1.13, P=0.28>0.05)、(RR=1.02, 95%CI:0.89-1.16, P=0.81>0.05)、(RR=1.00, 95%CI:0.95-1.05, P=0.90>0.05)、(RR=1.00, 95%CI:0.94-1.07, P=0.95>0.05)、(RR=0.99, 95%CI:0.92-1.07, P=0.83>0.05) (**Figure 6**).

### 3.5 Publication bias

**Figure 7:**
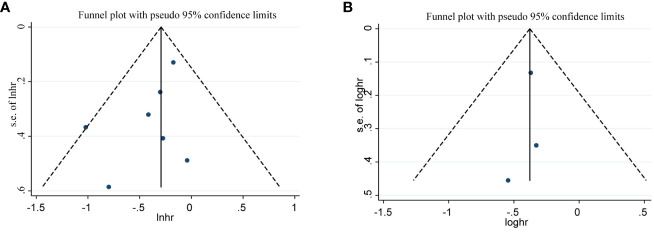
Funnel plot for OS **(A)**, PFS **(B)**.

**Figure 8:**
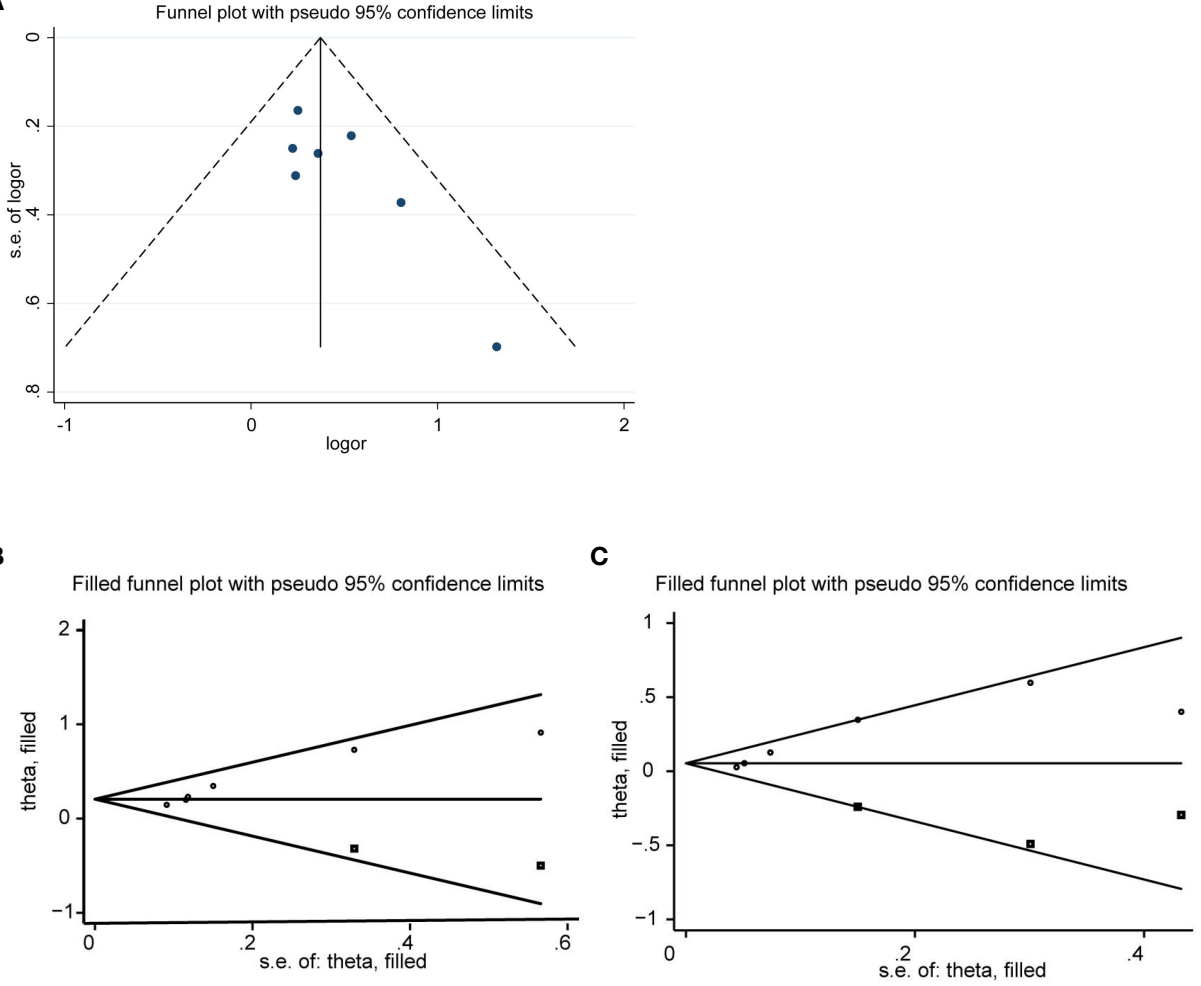
Funnel plot for CRR **(A)**, ORR **(B)**, DCR **(C)**.

We evaluated publication bias by visually observing the symmetry of the funnel plot and quantitatively Egger testing. Funnel plots of OS, PFS, CRR, no significant asymmetry was observed from visual assessment (**Figures 7A, B**, **8A**), and there was no publication bias using Egger testing (OS P = 0.180, PFS P = 0.659, CRR P = 0.067). The funnel plots of ORR and DCR showed asymmetry, and passed the Egger test, and there was publication bias (P=0.003<0.05, p=0.013<0.05). Therefore, the trim and fill method were used to assess whether publication bias in this meta-analysis affected the stability of the results. The updated results did not reverse (ORR:RR=1.229, 95%CI:1.077-1.427, p=0.003<0.05;DCR:RR=1.063, 95%CI:0.950-1.189, p=0.286>0.05), and the adjusted funnel chart was clearly symmetrical (**Figures 8B, C**). Therefore, the results of our meta-analysis are reliable.

## 4 Discussion

The poor prognosis of patients with LA-SCCHN drives the evaluation of CRT. In a meta-analysis of 107 randomized trials and 19805 patients (23), confirming that CRT was the primary treatment option for LA-SCCHN. However, even after CRT, local recurrence or distant metastasis remains a challenge. Related studies have found that the addition of antibodies targeting EGFR to CRT can improve treatment outcomes. However, many toxic deaths were reported in the combination therapy based on cetuximab or panitumumab (24–27), and overlapping toxicities hinder the implementation of the treatment. For example, According to adverse event reports from Cetuximab treatment, the incidence of adverse events is as high as 89%, including fatal pulmonary embolism and anaphylactic shock (28). And unlike the above EGFRMab, nimotuzumab would selectively target tumor cells with high expression of EGFR receptor without binding to normal tissues. Therefore, the incidence of adverse reactions is low (29, 30). At present, nimotuzumab has been widely used in the treatment of glioma (31), lung squamous cell carcinoma (32), esophageal cancer (33), pancreatic cancer (34) and other solid tumors. And showed a better efficacy and safety profile. In recent years, there have been many clinical studies of nimotuzumab in the treatment of SCCHN. A study by Reddy et al (19) evaluating the treatment of LA-SCCHN with nimotuzumab combined with RT/CRT showed that compared with RT alone, the 5-year OS of nimotuzumab combined with RT increased by 13% and the risk of death decreased by 24%; Compared with CRT, the 5-year OS of nimotuzumab combined with CRT increased by 27% and the risk of death decreased by 64%.Ang et al (35) single arm, Phase II study of nimotuzumab combined with CRT in patients with LA-SCCHN showed a CRR of 59%, a median PFS of 17.5 months (95% CI:11.1-54.5), a 3-year PFS of 40.4% (95% CI:24.3-55.9), no grade 3-4 skin toxicity, and the incidence of grade 3 or higher grade mucositis and hypokalemia was similar as expected for CRT. Another study of nimotuzumab plus chemotherapy for recurrent or metastatic head and neck squamous cell carcinoma (R/M SCCHN) showed that the overall response rate increased by 19.2% compared with chemotherapy alone (p = 0.023). Median PFS increased by 2 months (p = 0.009) without any significant increase in toxicity (36). Other studies of nimotuzumab in SCCHN (37, 38) also showed similar results. It can be seen that the treatment of nimotuzumab combined with chemotherapy or RT or CRT for SCCHN can improve the short-term and long-term efficacy without increasing the toxic reaction. Nimotuzumab has become a new option for the treatment of SCCHN. However, the sample size of these studies was small, so we considered using meta-analysis to study the efficacy and safety of nimotuzumab plus RT/CRT in LA-SCCHN.

This study searched the relevant clinical research literature and included 7 randomized controlled trials involving 1012 cases. The main purpose of this study was to evaluate the effect of combined treatment with nimotuzumab on OS and PFS. The results showed that compared with RT/CRT group, the combined treatment group with nimotuzumab had longer OS (HR=0.75,95%CI, 0.62-0.90, P<0.05) and better PFS (HR=0.69, 95% CI, 0.54-0.87, P<0.05). At the same time, the recent efficacy such as CRR, ORR, DCR was also analyzed, and the results showed that CRR and ORR were also significantly improved compared with RT/CRT alone. In addition, we analyzed the incidence of adverse effects after combination therapy and showed that only the nimotuzumab combination group was higher than the RT/CRT alone group in the incidence of rash, and there was no clear difference between the remaining adverse effects (neutropenia, anemia, nausea/vomiting, mucositis, dermatitis, dysphagia). But Patil et al (17) and Thai et al (16) showed that the incidence of rash was within a controllable range, and there was no rash incidence above grade 3. Therefore, this meta-analysis demonstrated controllable safety of combination therapy. Of course, there are some limitations in this meta-analysis: only 7 documents are included, and the sample size is not large enough; The quality score of the documents is uneven; There are differences in radiotherapy methods and chemotherapy doses among the study groups, and there are two control studies that only use radiotherapy because patients cannot tolerate chemotherapy drugs, which may lead to publication bias. Therefore, the conclusions in this paper need to be further demonstrated by more large samples and multi-center randomized controlled trials.

## 5 Conclusion

In this meta-analysis, we observed that OS, PFS, CRR, ORR were superior to RT/CRT in nimotuzumab plus RT/CRT with no significant difference in safety. This provides some evidence suggesting that adding nimotuzumab to RT/CRT may be an effective method for treating LA-SCCHN. However, more prospective randomized controlled clinical trials are still needed to fully explore the effectiveness of this treatment in patients with LA-SCCHN.

## Data availability statement

The original contributions presented in the study are included in the article/supplementary material. Further inquiries can be directed to the corresponding authors.

## Author contributions

MG: Writing – original draft. DZ: Writing – original draft, Writing – review & editing. YZ: Data curation, Methodology, Project administration, Writing – original draft. MM: Data curation, Project administration, Validation, Writing – original draft. KS: Methodology, Data curation, Writing – original draft. XW: Project administration, Formal analysis, Methodology, Supervision, Writing – original draft. CB: Formal analysis, Methodology, Supervision, Writing – original draft.

## References

[1] Siegel RL Miller KD Fuchs HE Jemal A . Cancer statistics, 2022. CA Cancer J Clin. (2022) 72:7–33. doi: 10.3322/caac.21708 35020204

[2] Rivera F Vega-Villegas ME Lopez-Brea MF Marquez R . Current situation of panitumumab, matuzumab, nimotuzumab and zalutumumab. Acta Oncol. (2008) 47:9–19. doi: 10.1080/02841860701704724 18097777

[3] Argiris A Karamouzis MV Raben D Ferris RL . Head and neck cancer. Lancet. (2008) 371:1695–709. doi: 10.1016/S0140-6736(08)60728-X PMC7720415 18486742

[4] Crombet Ramos T Mestre Fernandez B Mazorra Herrera Z Iznaga Escobar NE . Nimotuzumab for patients with inoperable cancer of the head and neck. Front Oncol. (2020) 10:817. doi: 10.3389/fonc.2020.00817 32537431 PMC7266975

[5] Concu R Cordeiro MNDS . Looking for new inhibitors for the epidermal growth factor receptor. Curr Top Med Chem. (2018) 18:219–32. doi: 10.2174/1568026618666180329123023 29595111

[6] Santini J Formento JL Francoual M Milano G Schneider M Dassonville O . Characterization, quantification, and potential clinical value of the epidermal growth factor receptor in head and neck squamous cell carcinomas. Head Neck. (1991) 13:132–9. doi: 10.1002/hed.2880130209 2022478

[7] Bianco C Tortora G Bianco R Caputo R Veneziani BM Caputo R . Enhancement of antitumor activity of ionizing radiation by combined treatment with the selective epidermal growth factor receptor-tyrosine kinase inhibitor ZD1839 (Iressa). Clin Cancer Res. (2002) 8:3250–8. 12374696

[8] Morales AA Duconge J Alvarez-Ruiz D Becquer-Viart ML Nunez-Gandolff G Fernandez E . Humanized versus murine anti-human epidermal growth factor receptor monoclonal antibodies for immunoscintigraphic studies. Nucl Med Biol. (2000) 27:199–206. doi: 10.1016/S0969-8051(99)00094-3 10773550

[9] Cai WQ Zeng LS Wang LF Wang YY Cheng JT Zhang Y . The latest battles between EGFR monoclonal antibodies and resistant tumor cells. Front Oncol. (2020) 10:1249. doi: 10.3389/fonc.2020.01249 32793499 PMC7393266

[10] Mazorra Z Chao L Lavastida A Sanchez B Ramos M Iznaga N . Nimotuzumab: beyond the EGFR signaling cascade inhibition. Semin Oncol. (2018) 45:18–26. doi: 10.1053/j.seminoncol.2018.04.008 30318080

[11] Rojo F Gracias E Villena N Cruz T Corominas JM Corradino I . Pharmacodynamic trial of nimotuzumab in unresectable squamous cell carcinoma of the head and neck: a SENDO Foundation study. Clin Cancer Res. (2010) 16(8):2474–82. doi: 10.1158/1078-0432.CCR-09-3042 20371675

[12] Yuan Y Chen J Fang M Guo Y Sun X Yu D . Nimotuzumab combined with chemoradiotherapy for the treatment of cervical cancer: A meta-analysis of randomized controlled trials. Front Oncol. (2022) 12:994726. doi: 10.3389/fonc.2022.994726 36263226 PMC9573994

[13] Ramakrishnan MS Eswaraiah A Crombet T Piedra P Saurez G Iyer H . Nimotuzumab, a promising therapeutic monoclonal for treatment of tumors of epithelial origin. MAbs. (2009) 1(1):41–8. doi: 10.4161/mabs.1.1.7509 PMC2715181 20046573

[14] Tierney JF Stewart LA Ghersi D Burdett S Sydes MR . Practical methods for incorporating summary time-to-event data into meta-analysis. Trials. (2007) 8:16. doi: 10.1186/1745-6215-8-16 17555582 PMC1920534

[15] Higgins JP Altman DG Gøtzsche PC Jüni P Moher D Oxman AD . The Cochrane Collaboration's tool for assessing risk of bias in randomised trials. BMJ. (2011) 343:d5928. doi: 10.1136/bmj.d5928 22008217 PMC3196245

[16] Thai Hoa NT Quang Huy H . Combined nimotuzumab with chemoradiotherapy for locally advanced head and neck squamous cell carcinoma. Cureus. (2020) 12:e8105. doi: 10.7759/cureus.8105 32426197 PMC7228796

[17] Patil VM Noronha V Joshi A Agarwal J Ghosh-Laskar S Budrukkar A . A randomized phase 3 trial comparing nimotuzumab plus cisplatin chemoradiotherapy versus cisplatin chemoradiotherapy alone in locally advanced head and neck cancer. Cancer. (2019) 125:3184–97. doi: 10.1002/cncr.32179 31150120

[18] Kumar A Chakravarty N Bhatnagar S Chowdhary GS . Efficacy and safety of concurrent chemoradiotherapy with or without Nimotuzumab in unresectable locally advanced squamous cell carcinoma of head and neck: Prospective comparative study - ESCORT-N study. South Asian J Cancer. (2019) 8(2):108–11. doi: 10.4103/sajc.sajc_38_18 PMC6498712 31069191

[19] Reddy BK Lokesh V Vidyasagar MS Shenoy K Babu KG Shenoy A . Nimotuzumab provides survival benefit to patients with inoperable advanced squamous cell carcinoma of the head and neck: a randomized, open-label, phase IIb, 5-year study in Indian patients. Oral Oncol. (2014) 50:498–505. doi: 10.1016/j.oraloncology.2013.11.008 24613543

[20] Rodriguez MO Rivero TC del Castillo Bahi R Muchuli CR Bilbao MA Vinageras EN . Nimotuzumab plus radiotherapy for unresectable squamous-cell carcinoma of the head and neck. Cancer Biol Ther. (2010) 9:343–9. doi: 10.4161/cbt.9.5.10981i 20448462

[21] Zhu Q Zhang Z . Nimotuzumab combined with concurrent radiotherapy and chemotherapy for locally advanced head and neck. Analysis of curative effect of squamous cell carcinoma. J China Med Univ. (2021) 50(6):556–9.

[22] Wu H Jin S Shen Q Sun R . Clinical observation of concurrent radiotherapy and chemotherapy for locally advanced pharyngolaryngeal cancer in nimotuzumab. Family medicine; See a doctor and choose medicine. (2020) (8):46–7.

[23] Lacas B Carmel A Landais C Wong SJ Licitra L Tobias JS . Meta-analysis of chemotherapy in head and neck cancer (MACH-NC): An update on 107 randomized trials and 19,805 patients, on behalf of MACH-NC Group. Radiother Oncol. (2021) 156:281–93. doi: 10.1016/j.radonc.2021.01.013 PMC8386522 33515668

[24] Mazorra Z Lavastida A Concha-Benavente F Valdes A Srivastava RM Garcia-Bates TM . Nimotuzumab induces NK cell activation, cytotoxicity, dendritic cell maturation and expansion of EGFR-specific T cells in head and neck cancer patients. Front Pharmacol. (2017) 8:382. doi: 10.3389/fphar.2017.00382 28674498 PMC5474456

[25] Ang KK Zhang Q Rosenthal DI Nguyen-Tan PF Sherman EJ Weber RS . Randomized phase III trial of concurrent accelerated radiation plus cisplatin with or without cetuximab for stage III to IV head and neck carcinoma: RTOG 0522. J Clin Oncol. (2014) 32(27):2940–50. doi: 10.1200/JCO.2013.53.5633 PMC4162493 25154822

[26] Hurt CN Nixon LS Griffiths GO Al-Mokhtar R Gollins S Staffurth JN . SCOPE1: a randomised phase II/III multicentre clinical trial of definitive chemoradiation, with or without cetuximab, in carcinoma of the oesophagus. BMC Cancer. (2011) 11:466. doi: 10.1186/1471-2407-11-466 22035459 PMC3212828

[27] Philip PA Benedetti J Corless CL Wong R O'Reilly EM Flynn PJ . Phase III study comparing gemcitabine plus cetuximab versus gemcitabine in patients with advanced pancreatic adenocarcinoma: Southwest Oncology Group-directed intergroup trial S0205. J Clin Oncol. (2010) 28(22):3605–10. doi: 10.1200/JCO.2009.25.7550 PMC2917315 20606093

[28] Stanbouly D Philipone E Morlandt AB Kaleem A Chuang S-K Patel N . Adverse events secondary to cetuximab therapy in head & neck cancer therapy and risk factors for serious outcomes. Oral Oncol. (2022) 131:105952. doi: 10.1016/j.oraloncology.2022.105952 35717723

[29] Garrido G Tikhomirov IA Rabasa A Yang E Gracia E Iznaga N . Bivalent binding by intermediate affinity of nimotuzumab: a contribution to explain antibody clinical profile. Cancer Biol Ther. (2011) 11(4):373–82. doi: 10.4161/cbt.11.4.14097 21150278

[30] Li J Yan H . Skin toxicity with anti-EGFR monoclonal antibody in cancer patients: a meta-analysis of 65 randomized controlled trials. Cancer Chemother Pharmacol. (2018) 82:571–83. doi: 10.1007/s00280-018-3644-2 30006755

[31] Massimino M Biassoni V Miceli R Schiavello E Warmuth-Metz M Modena P . Results of nimotuzumab and vinorelbine, radiation and re-irradiation for diffuse pontine glioma in childhood. J Neurooncol. (2014) 118(2):305–12. doi: 10.1007/s11060-014-1428-z 24696052

[32] Qiu B Wang D Li Q Wu Y Guo S Jiang X . Concurrent chemoradiation therapy with or without nimotuzumab in locally advanced squamous cell lung cancer: A phase 2 randomized trial. Int J Radiat Oncol Biol Phys. (2021) 111(4):917–25. doi: 10.1016/j.ijrobp.2021.06.032 34229051

[33] de Castro Junior G Segalla JG de Azevedo SJ Andrade CJ Grabarz D de Araújo Lima França B . A randomised phase II study of chemoradiotherapy with or without nimotuzumab in locally advanced oesophageal cancer: NICE trial. Eur J Cancer. (2018) 88:21–30. doi: 10.1016/j.ejca.2017.10.005 29179134

[34] Schultheis B Reuter D Ebert MP Siveke J Kerkhoff A Berdel WE . Gemcitabine combined with the monoclonal antibody nimotuzumab is an active first-line regimen in KRAS wildtype patients with locally advanced or metastatic pancreatic cancer: a multicenter, randomized phase IIb study. Ann Oncol. (2017) 28(10):2429–35. doi: 10.1093/annonc/mdx343 28961832

[35] Ang MK Montoya JE Tharavichitkul E Lim C Tan T Wang LY . Phase II study of nimotuzumab (TheraCim-hR3) concurrent with cisplatin/radiotherapy in patients with locally advanced head and neck squamous cell carcinoma. Head Neck. (2021) 43(5):1641–51. doi: 10.1002/hed.26635 33547683

[36] Yadav A Goyal P Agrawal CR Bothra SJ Jain P Choudhury KD . Efficacy and tolerability of nimotuzumab in combination with chemotherapy in recurrent and metastatic squamous cell carcinoma of head and neck at a cancer center in Northern India. Indian J Cancer. (2020) 57(1):76–83. doi: 10.4103/ijc.IJC_469_18 32129298

[37] Rawat S Tandan H Patel S Chaudhari S . Safety and efficacy of nimotuzumab with concurrent chemoradiotherapy in unresectable locally advanced squamous cell carcinoma of head and neck: An Indian rural hospital experience. South Asian J Cancer. (2019) 8:52–6. doi: 10.4103/sajc.sajc_76_18 PMC6348788 30766856

[38] Srinivas KS Sundaram R Divyambika CV Chaudhari S . Nimotuzumab with intensity-modulated radiation therapy in unresectable and platinum-ineligible locally advanced head-and-neck cancer. South Asian J Cancer. (2020) 9(1):43–6. doi: 10.4103/sajc.sajc_29_19 PMC6956585 31956621

